# The Role of Lung Resident Mesenchymal Stromal Cells in the Pathogenesis and Repair of Chronic Lung Disease

**DOI:** 10.1093/stmcls/sxad014

**Published:** 2023-02-07

**Authors:** Declan F Doherty, Lydia Roets, Anna D Krasnodembskaya

**Affiliations:** Wellcome-Wolfson Institute for Experimental Medicine, School of Medicine, Dentistry and Biomedical Sciences, Queen’s University Belfast, Belfast, UK; Wellcome-Wolfson Institute for Experimental Medicine, School of Medicine, Dentistry and Biomedical Sciences, Queen’s University Belfast, Belfast, UK; Wellcome-Wolfson Institute for Experimental Medicine, School of Medicine, Dentistry and Biomedical Sciences, Queen’s University Belfast, Belfast, UK

**Keywords:** mesenchymal stromal cells, chronic lung disease, idiopathic pulmonary fibrosis, chronic obstructive pulmonary disease, bronchopulmonary dysplasia, bronchiolitis obliterans

## Abstract

Mesenchymal stromal/stem cells are multipotent adult cells that can be extracted from numerous tissues, including the lungs. Lung-resident MSCs (LR-MSCs) are localized to perivascular spaces where they act as important regulators of pulmonary homeostasis, mediating the balance between lung injury/damage and repair processes. LR-MSCs support the integrity of the lung tissue via modulation of the immune response and release of trophic factors. However, in the context of chronic lung diseases, the ability of LR-MSCs to maintain pulmonary homeostasis and facilitate repair is diminished. In this setting, LR-MSC can contribute to the pathogenesis of disease, through their altered secretory and immunomodulatory properties. In addition, they are capable of differentiating into myofibroblasts, thereby contributing to the fibrotic aspects of numerous lung diseases. For example, in idiopathic pulmonary fibrosis, a variety of factors can stimulate their differentiation into myofibroblasts including tumor necrosis factor-α (TNF-(α), transforming growth factor-β1 (TGF-β1), endoplasmic reticulum (ER) stress, Hedgehog (HH), and Wingless/integrated (Wnt) signaling. Here, we review the current literature on the characterization of LR-MSCs and describe their roles in pulmonary homeostasis/repair and in the pathogenesis of chronic lung disease.

## Graphical Abstract

**Graphical Abstract:**
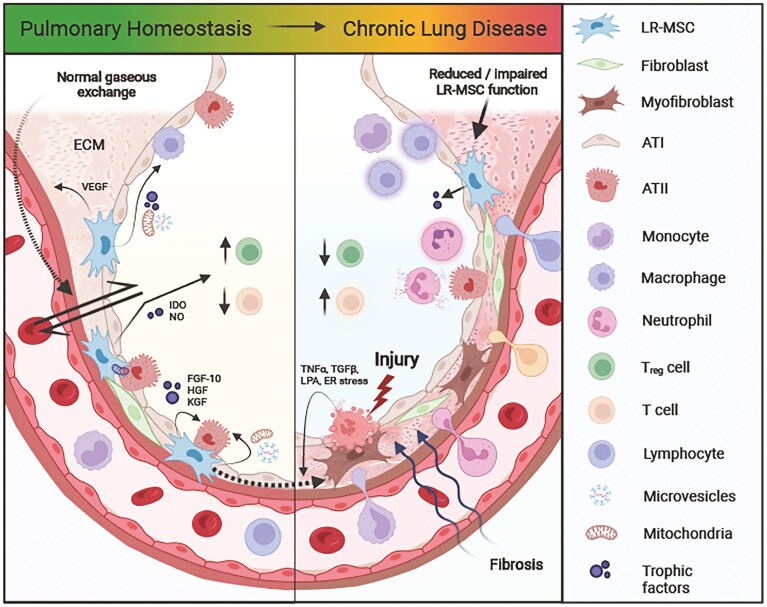

**Significance Statement**

Mesenchymal stromal/stem cells have shown great promise as a therapy for lung diseases. Current literature has predominantly focused on the therapeutic effects of exogenously administered MSCs derived from tissue sources such as the bone marrow, adipose tissue, or umbilical cord; whereas much less is known about the function and utility of endogenous lung-resident MSCs. A better understanding of the homeostatic and pathogenic roles played by LR-MSCs is important for improving our knowledge of disease pathogenesis and should prove beneficial as we move closer to the development of a clinically efficacious cellular/cell product therapy. This review summarizes the role of lung-resident MSCs in the regulation of pulmonary homeostasis/repair and their involvement in the pathogenesis of various chronic lung diseases.

## Introduction

Mesenchymal stromal/stem cells (MSCs) are spindle-shaped multipotent cells that are found in the mesenchymal stroma or connective tissues of the body.^1^ They serve as important regulators of tissue homeostasis and regeneration by (A) differentiating into essential cell types, (B) extracellular matrix deposition, (C) direct cell-to-cell contact, and (D) the secretion of paracrine mediators.^2,3^ First isolated from the bone marrow in 1970 by Friedenstein et al,^4^ human MSCs can now be derived from a variety of additional sources, including the lungs.^5^ MSCs function to support tissue integrity, promote tissue repair,^6^ and modulate the immune response.^7^ They were first tested as a cellular therapy in humans in 1995.^8^ Since then, they have been the focus of intense global efforts to treat disorders with unmet clinical needs, including COVID-19.^9^ Although the vast majority of studies reported in the literature focus on the therapeutic application of exogenous bone marrow-derived (BM-)MSCs for respiratory pathologies, there is a growing interest in the potential of lung-resident MSCs (LR-MSCs) to help identify more targeted treatments.^10^ This is unsurprising given the importance of diffusible signals from the lung mesenchyme for pulmonary development (eg, early patterning and morphogenesis, and alveolarization) and maintenance.^11^ This concise review focuses on the roles of LR-MSCs in the pathogenesis and repair of various chronic lung diseases. The role of LR-MSCs in lung cancer is outside the scope of this article and so we instead refer the reader to a recent review on this topic.^12^

## Lung Resident MSCs

### Classification and Location

At present, MSCs are characterized according to the criteria proposed by The International Society for Cellular Therapy (ISCT): the cells must have a positive expression of CD73, CD90, and CD105; while lacking immune/hematopoietic markers such as CD14, CD34, CD45, and HLA-DR surface molecules.^1,13^ More recently, another ISCT position statement paper described its support for the acronym “MSCs” but recommended it be supplemented with the tissue source of the cells, evidence of in vitro and in vivo stemness, and functional characterization to demonstrate MSC properties.^1,14^ Furthermore, MSCs must be plastic-adherent while cultured under standard conditions and they should have the capacity to differentiate into mesodermal cell types such as adipocytes, chondroblasts, and osteoblasts in vitro.^13^ Interestingly, results from in vitro and in vivo studies have hinted at the trans-differentiation potential of MSCs into cell types of ectodermal and endodermal lineages, via mesenchymal-to-epithelial transition.^15,16^ However, the lack of consensus regarding what exactly constitutes a LR-MSC, combined with non-standardized isolation techniques and cultivation methods has led to ambiguous findings. For instance, the similarity between MSCs and fibroblasts has caused confusion around whether they are distinct cell lineages or are instead different phases along a continuous scale of differentiation, particularly as both cell types may contribute to fibrotic diseases via their differentiation to myofibroblasts.^17^ Fibroblasts are also characterized as plastic-adherent with a similar mesodermal differentiation capacity (to MSCs) and possess their own immunoregulatory properties.^18,19^ Instead of looking for the presence or absence of cell surface markers that are largely non-specific, comparing the expression levels of certain surface proteins may help better distinguish between the 2 cell types. Indeed, MSCs have been documented to exhibit higher expression of CD106 with lower expression of CD9 relative to fibroblasts; however, expression of these markers was found to converge with passaging toward the levels observed in fibroblasts suggesting that fibroblasts are aged MSCs.^20^ Given the aforementioned similarities, it is possible that the MSC and fibroblast populations studied in vitro may be heterogeneous and a mixture of both, since they are not easily separated.^21^ Details documenting a working definition of terms used to describe the main mesenchymal cells discussed in the text can be found in Table 1. In addition, details of the isolation method and characterization of LR-MSCs used for the references discussed in this review have been included in Table 2.

**Table 1.** Definitions of the mesenchymal cell nomenclature used in the review.

Term	Definition	Reference(s)
Mesenchymal stromal cell (MSC)	Non-hematopoietic, multipotent, self-renewing mesenchymal stromal cells are found in various tissues of the body. MSCs are plastic adherent and have a positive surface expression of CD73, CD90, and CD105, whilst negative for immune and endothelial cell surface markers (including CD45, CD34, CD14, or CD11b, CD79alpha or CD19, and HLA-DR), and the ability to differentiate into osteoblasts, adipocytes, and chondroblasts in vitro	^13^
Mesenchymal stem cell	Cells meeting the criteria for mesenchymal stromal cells, in addition to demonstrable progenitor cell functionality using both in vitro and in vivo techniques	^1^
Lung resident-MSC (LR-MSC)	Cells meeting the criteria for mesenchymal stromal cells which have been isolated specifically from lung tissue or fluid collected from lung tissue	^1^
Lung pericyte	Multipotent perivascular cells that are embedded in the basement membrane of pulmonary and systemic capillaries and express markers including PDGFR-β and NG2+	^22^
Mesenchymal progenitor cell (MPC)	Progenitor cells give rise to all mesenchymal lineages in the lung. These multipotent mesenchymal progenitors are characterized as Gli1+ Wnt2+ Isl1+ expressing in embryonic tissue but express various markers in the adult lung depending on location	^23,24^

**Table 2.** Summary of the isolation techniques and characterization methods for LR-MSCs carried out by the studies described in this review.

Reference(s)	Source	Isolation method	Plastic adherence	Phenotype (postitive)			Phenotype (negative)					In vitro differentiation			Surface phenotype Additional markers	CFU-F
				CD73	CD90	CD105	CD34	CD45	CD14/ CD11b	CD19/ CD79a	HLA-DR	Osteo	Adipo	Chondro		potential
ISCT minimal criteria for defining MSCs	Human		✓	✓	✓	✓	✓	✓	✓	✓	✓	✓	✓	✓		
^5^	Human	Tissue digestion	✓	✓	✓	✓	✓	✓				✓	✓	✓	CD31−, CD166+, STRO-1	
^25^	Mouse	Tissue digestion	✓	✓	✓	✓		✓				✓		✓	CD31−, EpCAM−, CD146+, SCA1+	✓
^28^	Human	Tissue digestion	✓	✓	✓	✓	✓	✓	✓	✓	✓	✓	✓	✓	CD31−, CD44+, SSEA4+	✓
^29^	Human	Tissue digestion	✓	✓	✓	✓	✓	✓	✓	✓	✓	✓	✓	✓	CD31−, CD271−, CD146+, HLA Class I+	✓
^57^	Human	Tissue digestion	✓	✓	✓	✓		✓				✓	✓	✓		✓
^58^	Human	Tissue digestion	✓												SSEA4+	
^37^	Mouse	Tissue digestion	✓	✓	✓	✓		✓				✓	✓	✓	F480−, c-kit−, CD146−, CD44+, CD106+, CD133+, Sca1+	
^38^	Human & mouse	Tissue digestion	✓	✓		✓		✓	✓						CD31−, CD106-lo, CD140a-lo, CD140b-hi, CD44+, ABCG2+	✓
^61^	Mouse	Gli1+ FACS	✓			✓	✓	✓				✓	✓	✓	CD31−, CD29+, Sca1+, CD44+	✓
^62^	Human and mouse	Gli1+ FACS						✓							CD31−, CD45−, CD325a-, CD326−, Gli1+	
^63^	Human and mouse	Tissue digestion	✓		✓	✓	✓	✓				✓	✓	✓	CD31−, Sca1+	
^64^	Mouse	Tissue digestion	✓				✓	✓							CD31−, CD29+, Sca1+	
^65^	Mouse	Tissue digestion	✓				✓	✓							CD31−, CD29+, Sca1+	
^66^	Human	BAL fluid	✓	✓	✓	✓	✓	✓	✓			✓	✓	✓	CD44+	✓
^67^	Human	BAL fluid	✓	✓	✓	✓	✓	✓	✓			✓	✓	✓		✓
^39^	Human and mouse	Tissue digestion	✓	✓		✓	✓	✓			✓	✓	✓		CD31−, CD44+, HLA−ABC+, CD29+, CD106+, Sca1+	
^68^	Mouse	Tissue digestion	✓		✓			✓							CD31−, CD29+, CD44+, CD106+, Sca1+	
^72^	Human	Tissue digestion	✓	✓	✓	✓	✓	✓				✓	✓	✓	CD31−, CD13+, CD29+, CD44+	
^73^	Human	Tissue digestion	✓	✓	✓	✓	✓	✓				✓	✓		CD31−, CD29+, CD44+, CD146+	✓
^75^	Human	Tissue digestion	✓	✓	✓	✓	✓	✓			✓	✓	✓	✓	CD14−, CD19−, CD146+	✓
^76^	Human	Tracheal aspirate	✓	✓	✓	✓	✓	✓	✓			✓	✓		CD31−, CD13+, CD166+	✓
^77^	Rat	Tissue digestion	✓	✓	✓		✓	✓	✓			;	✓	✓	MHC class II RT1B−, CD146+, CD166+	✓
^78^	Rabbit	Tissue digestion	✓						✓	✓	✓	✓	✓	✓	CD117−, CD44+, CD81+	✓
^40^	Human	BAL fluid	✓	✓	✓	✓		✓				✓	✓		CD44+	
^79^	Human	BAL fluid	✓	✓		✓	✓	✓	✓			✓	✓	✓	Vimentin+	✓
^80^	Human	BAL fluid	✓	✓	✓	✓	✓	✓	✓		✓	✓	✓	✓	HLA-DQ−, HLA-1+	✓
^81^	Human	BAL fluid	✓												CD34−, CD45−, CD44+, CD90+, CD105+ (data not shown)	
^82^	Mouse	Tissue digestion	✓	✓	✓	✓	✓	✓				✓	✓		CD31−, Sca-1+, Stro-1+	✓
^89^	Mouse	Tissue digestion	✓	✓	✓	✓		✓	✓						CD34+	
^90^	Rat	BAL fluid	✓	✓	✓		✓	✓				✓	✓	✓	CD29+	✓
^92^	Human	BAL fluid	✓	✓	✓	✓	✓	✓				✓	✓	✓	CD14−	✓
^45^	Rat	BAL fluid	✓	✓	✓		✓	✓				✓	✓	✓	CD29+	✓
^41^	Mouse	Tissue digestion	✓	✓	✓	✓	✓	✓	✓			✓	✓	✓	CD106−, CD44+, Sca1+	
^42^	Mouse	Tissue digestion	✓		✓			✓							CD31−, CD29+, CD44+, CD106+, Sca1+	

Abbreviations: ABCG2, ATP binding cassette subfamily G member 2; BAL, bronchoalveolar lavage fluid; CD, cluster of differentiation; EpCAM, epithelial cell adhesion molecule; Gli1, glioma-associated oncogene 1; HLA-DR, human leukocyte antigen-DR isotype; ISCT, International Society for Cellular Therapy; MHC class II, major histocompatibility complex; MSC, mesenchymal stromal/stem cell; SCA1, stem cells antigen-1; SSEA4, stage-specific embryonic antigen-4; STRO-1, mesenchyme 1.

The use of omics approaches to study tissue-specific MSCs has provided more insight into the characterization of LR-MSC populations. Indeed, scRNA-seq of murine fetal LR-MSCs—that were processed immediately after isolation to preserve their in vivo activation status—identified *Col14a1*, *Ly6a*, *Lum*, *Serpinf1*, and *Dcn* as markers of murine LR-MSC expressed both in situ and also following subsequent culture in vitro.^25^ Better characterization is particularly important as MSC-based medicinal products have diversified over the last decade.^26^ Indeed, MSCs isolated from different sources exhibit variable levels of incompatibility with human blood^27^ and so should LR-MSCs be developed as a clinical product that warrants intravascular delivery, the cells will need to be characterized for tissue factor (TF/CD142) surface expression and hemocompatibility after expansion.

**Figure 1.**
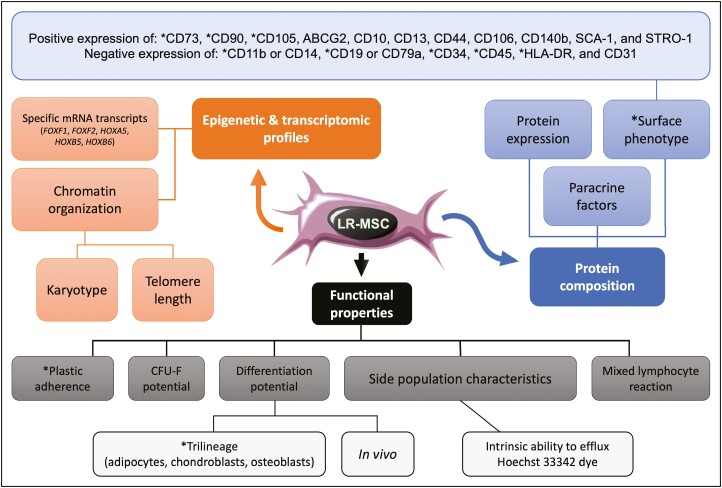
Different genomic, phenotypic, and functional properties for identifying high-quality human LR-MSCs. The original “minimal” criteria proposed to define primary human MSCs by the International Society for Cellular Therapy are marked with asterisks. Due to the macroscopic similarities between MSCs and fibroblasts, expanded criteria at the molecular and functional levels (eg, epigenetic modifications, specific transcriptomic profiles, telomere length, and side population characteristics) are crucial for identifying differences and enabling improved enrichment of “true (LR-) mesenchymal *stem* cells”. While karyotypic analysis is not required for routine identification of MSCs, it may be useful to verify a normal karyotype for extensively passaged or induced pluripotent stem cell-derived MSCs to minimize the occurrence of transforming events. Additional functional readouts, such as in vivo differentiation potentials and mixed lymphocyte reactions, along with the release of particular paracrine factors could be used to select MSCs for a tailored therapeutic response, ie, to elicit a reparative/regenerative response and/or to modulate an immune reaction. References.^1,4,13,29,37-46^.

**Figure 2.**
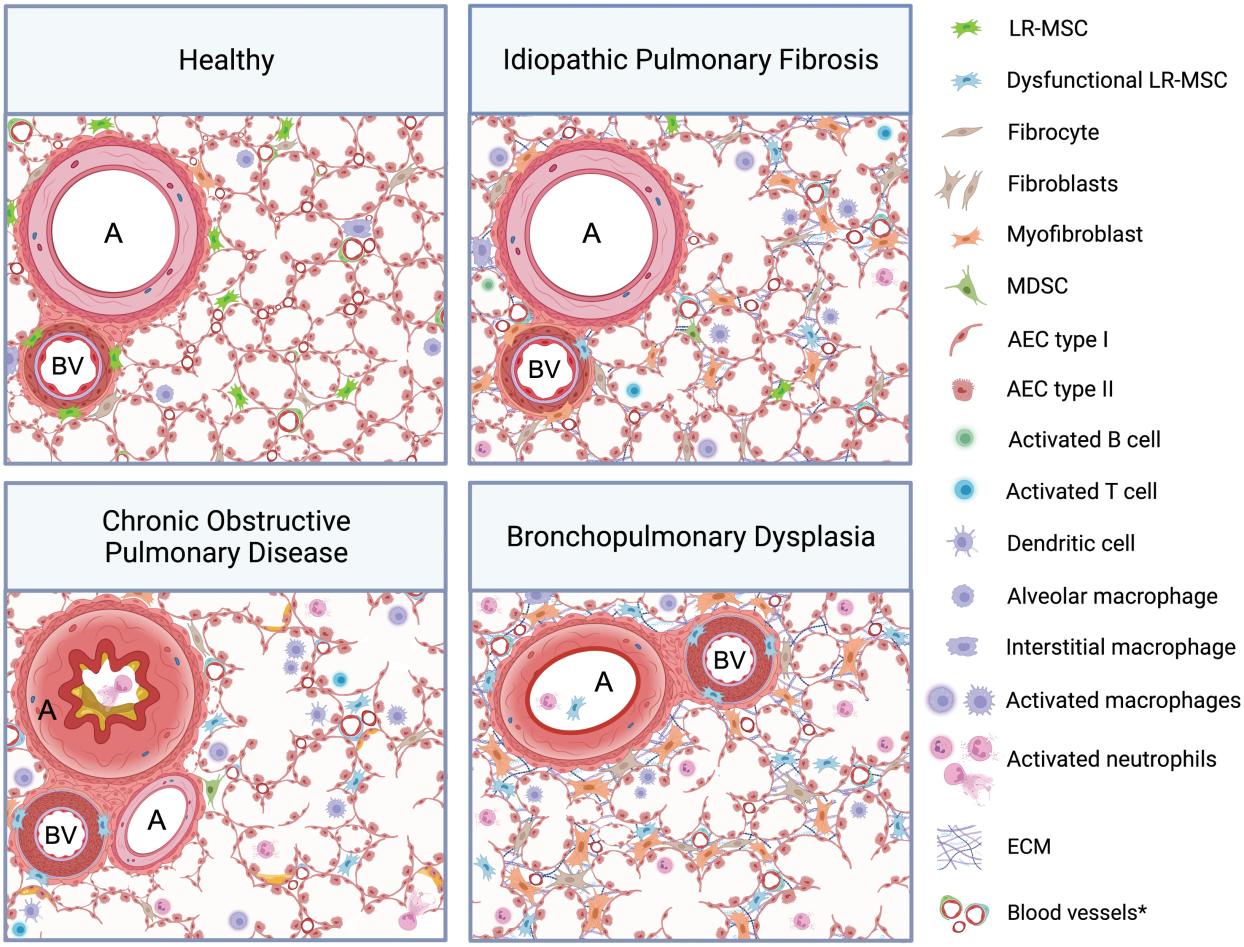
The differences in location and functionality of lung-resident MSCs during health and disease. (**A**) In healthy lungs, LR-MSCs can be found within the tunica adventitia or outermost layer of large and mid-sized blood vessels, as well as within the alveolar interstitium where they are closely apposed to type I alveolar epithelial cells. This ideally situates the cells for maintenance of pulmonary homeostasis, via a combination of local cell-to-cell communications, mitochondrial transfer, and paracrine production of immunomodulatory mediators. (**B**) Repetitive local micro-injuries to alveolar epithelial cells along with ineffective repair in the aging lung is believed to promote the differentiation of mesenchymal cells, including LR-MSCs, to pathogenic myofibroblasts which contribute to ECM deposition and IPF progression. This differentiation can be driven by multiple signaling pathways including but not limited to TNFα signaling via NF-κB, TGF-β1 signaling, the LPA-LPAR1 pathway, Wnt/β-catenin, and Hedgehog/GLI signaling. (**C**) LR-MSC functionality is altered in COPD. The cells exhibit defective immunomodulatory properties and diminished repair responses, characterized by impaired reduction of CD8+ T-cell proliferation and reduced production of HGF and FGF-10. This may play a role in the chronic airway inflammation, progressive airflow limitation, and emphysematous destruction of the lung parenchyma in COPD patients. (**D**) Hyperoxia and mechanical ventilation, used for the treatment of BPD, perturb LR-MSC functionality. The cells are abundant within fetal lungs and when isolated from tracheal aspirates of preterm infants, they may serve as a biomarker for BPD development. These LR-MSCs have diminished expression of growth factors involved in alveologenesis and repair, eg, VEGF and FGF-10. Abbreviations: A, airway; AEC, alveolar epithelial cell; BV, blood vessel; ECM, extracellular matrix; FGF-10, fibroblast growth factor-10; HGF, hepatocyte growth factor; IDO, indoleamine-2,3-dioxygenase; NO, nitric oxide; KGF, keratinocyte growth factor; LPA, lysophosphatidic acid; MDSC, myeloid-derived suppressor cell; PGE2, prostaglandin E2; TGF-β1, transforming growth factor-beta 1; TNF-α, tumor necrosis factor alpha; VEGF, vascular endothelial growth factor. *Blood vessels with and without (dys)functional pericytes/LR-MSCs. Created with BioRender.com.

We have outlined the ISCT’s “minimal” criteria for human MSCs along with our own expanded recommendations for identifying high-quality human LR-MSCs in Fig. 1. LR-MSCs appear to predominantly reside in the vascular stem cell niche within the adventitia (ie, the interface between the vessel wall and surrounding tissue) of large and mid-sized arteries and veins; whereas within the smallest blood vessels or capillaries, they can be found in the alveolar interstitium near endothelial cells that are closely apposed to sheet-like (type I) alveolar epithelial cells^28,29^ (Fig. 2A). The perivascular location of tissue-resident MSCs in fetal and adult human organs along with their in situ co-expression of both pericyte and MSC markers^22,25,30,31^ resulted in the somewhat controversial notion that MSCs correspond to pericytes or that pericyte serve as progenitors for tissue-resident MSCs.^32,33^ However, given the identification of MSC subpopulations at extravascular sites like the endosteum, it was counter-proposed that perivascular MSCs act as precursors of pericytes and other stromal cells under steady-state conditions.^34^ Interestingly, Feng et al discovered a dual origin of MSCs, ie, pericyte-derived and non-pericyte-derived within the murine incisor, leading them to posit that pericytes may not be the only cellular source of MSCs in different tissues; ergo, the tissue-specific extent of vascularity and growth/repair kinetics could account for the conflicting data regarding the contribution of pericyte-derived MSCs in different tissues.^35^ Nevertheless, it stands to reason that the perivascular location of LR-MSCs ideally situates the cells for the maintenance of pulmonary homeostasis.^36^

### Pulmonary Homeostasis

Pulmonary homeostasis is a delicate balance between repair/regeneration and the apoptotic, destructive, and inflammatory processes within the lungs. Maintenance of this balance is essential for the preservation of normal lung tissue and function. Particularly as the lungs are constantly exposed to harmful particulates circulating within the pulmonary vasculature as well as those that are inhaled from the surrounding atmosphere. Elaborate pulmonary defense mechanisms (ie, first-line filtration and removal systems) exist and, arguably, of equal or greater purpose is the immunosuppressive tone of the stromal environment, which regulates leukocyte recruitment and activation to prevent collateral (lung) damage.^18^

Due to the lack of literature outlining the precise mechanisms of action of human LR-MSCs, we have inferred their potential homeostatic functions from studies using MSCs derived from alternative sources, eg, murine LR-MSCs, BM-MSCs, induced pluripotent stem cell (iPSC)-derived MSCs. MSCs produce a wide array of immunosuppressive mediators—either directly themselves, or indirectly by inducing production in target cells. The soluble mediators (eg, indoleamine-2,3-dioxygenase, nitric oxide, and prostaglandin E2) can alter or inhibit the activity of innate and adaptive immune cells^2,47,48^ and can be secreted “spontaneously” or following induction of MSCs by pro-inflammatory cytokines—eg, IFN-γ and TNF-α.^49^ LR-MSCs also play a significant role in maintaining the integrity of the lungs by participating in epithelium-mesenchyme crosstalk via the production of various peptide growth factors, including the alveolar epithelial mitogen, fibroblast growth factor-10 (FGF-10).^50^ (BM-)MSCs have been found to prevent oxidative stress-induced lung damage by inhibiting reactive oxygen species (ROS),^51^ and they also secrete antimicrobial peptides—such as lipocalin-2^52^ and LL-37^53^—that protect against infections by direct antimicrobial action. Furthermore, (BM- and iPSC) MSCs are capable of transferring mitochondria to alveolar macrophages^54,55^ and epithelial cells^56^ alike, thereby enhancing the recipient cells’ bioenergetics. This shifts macrophage polarization toward an anti-inflammatory M2-like phenotype (demonstrated by enhanced phagocytosis and suppression of pro-inflammatory cytokine release) and helps mitigate lung injury. Indeed, mitochondrial transfer from LR-MSCs (derived from the digestion of normal human lung tissue) to BEAS2B cells (a human bronchial epithelial cell line) has been shown to be mediated through both contact-dependent and contact-independent mechanisms via microtubules/tunneling nanotubes and extracellular vesicles, respectively.^57^

The overarching role of LR-MSCs is to support nearby parenchymal cells, making them key regulators of pulmonary homeostasis. This is achieved via a combination of organelle transfer, the production of paracrine factors, and local cell-to-cell communications.

## Lung Resident MSCs in Pathogenesis of Chronic Lung Diseases

### Idiopathic Pulmonary Fibrosis (IPF)

IPF is a chronic, progressive, fibrotic, interstitial pneumonia that has a high mortality rate. Although there is an incomplete understanding of the pathogenesis of IPF, the modern hypothesis highlights repetitive local micro-injuries to alveolar epithelial cells (AECs) as central to development of the disease. In the aged lung, damage to AECs accompanied by ineffective repair causes the release of factors involved in the proliferation, activation, and differentiation of fibroblasts to matrix-producing myofibroblasts (Fig. 2B). These cells represent key effectors in lung fibrosis, which secrete extracellular matrix (ECM) proteins, increase tissue rigidity, cause progressive scarring, and destruction of lung architecture leading to impaired gaseous exchange, respiratory failure, and ultimately death.

Investigating the role of mesenchymal progenitor cells (MPC) from human IPF patients using single-cell RNA sequencing (scRNA-seq) revealed that these cells exist in a continuum between the undifferentiated state and differentiated fibroblasts.^23,24,58^ Using an embryonic determinant, stage-specific embryonic antigen 4 (SSEA4), to select the MPCs, allowed the identification of a hierarchical mesenchymal progenitor, which in IPF, was found to acquire a pathological phenotype at an early stage of its differentiation.^58^ These findings suggest that IPF fibroblasts acquire a pathologic role even at the earliest stages of their differentiation. Furthermore, a recent study using scRNA-seq of mouse and human pulmonary mesenchymal cells, suggested that fibrotic injury increases extracellular matrix (ECM) related genes in all mesenchymal cells not just myofibroblasts.^59^ Interestingly, in this study using gene expression signatures of mesenchymal cells, they did not detect evidence of differentiation to myofibroblasts from other mesenchymal populations.^59^ These findings suggest that although myofibroblasts are key effector cells in fibrosis, the whole mesenchymal cell population may respond to fibrotic injury and contribute to disease. Nonetheless, data from both animal models and human IPF patients reveal LR-MSCs as a source of the pathogenic myofibroblast population. In bleomycin-treated mice and patients with IPF, initial studies revealed the numbers of LR-MSCs were diminished due to their differentiation into myofibroblasts, which contribute to ECM deposition and disease progression.^37,38^ Indeed, bleomycin studies in mice have shown that platelet-derived growth factor receptor beta (PDGFRb)+/ neural/glial antigen 2 (NG2)+ pericytes give rise to ~45% of alpha smooth muscle actin (αSMA+) expressing interstitial myofibroblasts, akin to LR-MSCs.^60^ Comparable results were observed from a subsequent study that demonstrated glioma-associated oncogene homolog 1 (Gli1+) positive perivascular MSC-like cells contributed to ~37% of αSMA+ interstitial cells following bleomycin insult.^61^ More recently, in bleomycin-treated mice, Gli1+ MSC-like cells accounted for the entire myofibroblast subset of cells that generated scarring in the alveoli, as assessed using single-cell transcriptomic data.^62^ In addition to their pathogenic mechanism of myofibroblast differentiation, Gli1+ MSC-like cells also have a role in the promotion of metaplastic differentiation of epithelial progenitors in the airway, producing keratin 5 (KRT5+) basal cells.^62^ In fibrosis, Gli1+ MSC-like cells use Hedgehog signaling to block bone morphogenetic protein (BMP) signaling in epithelial progenitors to promote metaplasia, whereas restoring BMP signaling could reduce epithelial metaplasia and promote differentiation to ATII cells.^62^ In IPF, the metaplastic epithelia at sites of injury can contribute to scar formation associated with disease progression.

The differentiation of LR-MSCs to myofibroblasts results from a plethora of stimulatory signals within the microenvironment to which they are exposed and seems to involve multiple signaling pathways including Wnt/β-catenin and Hedgehog/GLI. In addition, ER stress has been identified within the IPF lung and has recently been linked to the differentiation of LR-MSCs to myofibroblasts, with the C/EBP homologous protein (CHOP) noted as being integral to this process.^63^

During the initial stages of IPF, there is activation of chronic inflammation and the release of pro-inflammatory factors such as IL-6 and TNF-α, as well as pro-fibrotic factors like TGF-β. TNF-α is one of the best-characterized inducers of the transcription factor nuclear factor kappa B (NF-κB), and both TNF-α and NF-κB signaling are upregulated in bleomycin-injured lung tissue.^64^ It was also noted that suppression of NF-κB signaling reduces the differentiation of LR-MSCs to myofibroblasts and diminishes lung fibrosis.^64^ These data suggest there is a link between inflammatory signaling in the promotion of LR-MSC differentiation to myofibroblasts. In addition, the pro-fibrotic cytokine TGF-β1 has been identified as having a role in LR-MSC differentiation to myofibroblasts. The major cellular sources of TGF-β1 in pulmonary fibrosis are alveolar macrophages and injured alveolar epithelial type II cells (ATII). TGF-β1 treatment of LR-MSCs causes enhanced expression of collagen I, fibronectin, and αSMA, all markers indicating differentiation to myofibroblasts, a process involving the upregulation of *miR-877-3p* expression.^65^ Smad7 (a target of *miR-877-3p*) is an inhibitory protein that halts TGF-β induced αSMA and collagen I expression. Thus, TGF-β1 promotes LR-MSC differentiation and reduces Smad-7 inhibitory pathways to promote fibrosis.

LR-MSCs express the lysophosphatidic acid (LPA) receptor 1 (LPAR1).^66^ During lung injury, the bioactive lipid LPA is released from numerous cells, which activates the LPAR1 receptor on LR-MSCs, causing the LR-MSCs to migrate and differentiate into fibroblasts. This process is dependent on the β-catenin pathway, where LPA enhances GSK3β phosphorylation, β-catenin nuclear translocation, and cellular migration.^66^ The LPA-LPAR1 pathway is crucial for fibroblast recruitment in IPF, and so LR-MSCs contribute to this pathomechanism of disease.

LR-MSCs from the terminal airways-alveoli can be obtained using bronchoalveolar lavage (BAL) fluid collected from human adult lungs. Characterization of LR-MSCs from the BAL fluid of patients with stable and progressive IPF have identified differential expression patterns in genes that regulate lung development, including FGF-10 and BMP-4.^67^ These findings highlight the suppression of the epithelial mitogen FGF-10 in progressive IPF and identified TGF-β and sonic hedgehog protein (SHH) signaling as critical mediators of this effect in LR-MSCs.

Expression levels of Wnt proteins are low in the normal adult lung but markedly elevated in IPF patients. Canonical Wnt signaling is activated after bleomycin instillation in mouse lungs and regulates the differentiation of LR-MSCs. Indeed, Wnt10a and Wnt8b are upregulated when LR-MSCs differentiate to myofibroblasts.^39,68^ Additionally, recombinant Shh stimulation promoted similar changes in LR-MSCs, highlighting a role for the Shh/Gli pathway in the myofibroblastic transition of LR-MSCs.^39^ Overexpression of the deSUMOylation enzyme SENP1 was observed in LR-MSCs differentiating into myofibroblasts.^63^ Downregulation of SENP1 could reverse this differentiation by promoting SUMOylation of Wnt and HH proteins, and enhancing the degradation of β-catenin and GLI1. The findings from these studies link HH and Wnt signaling with the differentiation and fibrotic effects of LR-MSCs.

### Chronic Obstructive Pulmonary Disease (COPD)

COPD is a multifaceted inflammatory disease that is a major cause of morbidity, mortality, and healthcare burden worldwide. COPD is caused by inhalation of noxious substances, with tobacco smoke being a major risk factor. The disease is characterized by chronic inflammation of the airways, accompanied by progressive and irreversible airflow limitation. Remodeling of the small airway compartment and loss of elastic recoil due to emphysematous destruction of the lung parenchyma results in the decline of lung function in COPD (Fig. 2C).

In animal models of emphysema, LR-MSCs have been shown to be effective when used as an exogenously administered therapeutic. While both LR- and BM-MSCs were found to reduce lung damage to a similar extent in a mouse model of elastase-induced emphysema, LR-MSCs were retained in the lungs for longer, presumably due to their higher expression of ICAM-1.^69^ These findings were supported by another independent study which assessed LR-MSCs (in addition to BM-MSCs and adipose-derived MSCs) in a similar murine model of elastase-induced emphysema. Intratracheal administration of LR-MSCs reduced the mean linear intercept, increased elastic fiber content within the lung parenchyma, and decreased collagen deposition around the small airways, while also limiting neutrophil infiltration and attenuating damage to type II airway epithelial and endothelial cells.^70^ A large animal study using LR-MSCs also revealed beneficial effects when transplanted endoscopically into sheep with experimental emphysema. During a follow-up 4 weeks later, transplants of LR-MSCs (5-10 × 10^6^ cells/site) on a scaffold matrix were found to be well tolerated with increased tissue mass and lung perfusion demonstrated over control animals.^71^ These findings were confirmed histologically, showing increased cellularity and ECM content in the lungs of LR-MSC-treated sheep.

LR-MSCs have been isolated from never-smokers and smokers in similar numbers, indicating that the reservoir of pulmonary MSCs in patients with COPD is not exhausted.^72^ LR-MSCs from current smokers with COPD elicited an impaired reduction of CD8+ T-cell proliferation. This was further confirmed in vitro, as cigarette smoke extract (CSE)-treated LR-MSCs demonstrated reduced T-cell immunomodulatory capacities.^72^ These findings suggest that the oxidative stress caused by smoking tobacco can impair the immunomodulatory capacity and homeostatic functions of LR-MSCs. Furthermore, COPD LR-MSCs exhibit a reduced ability to produce hepatocyte growth factor (HGF) and FGF-10, rendering them unable to orchestrate appropriate alveolar repair mechanisms^73^ (Fig. 2C). Given the therapeutic benefits of administering “healthy” LR-MSCs in pre-clinical models of emphysema/COPD, the data suggest that COPD LR-MSCs have reduced immunomodulatory functions and diminished repair responses.

### Bronchopulmonary Dysplasia (BPD)

BPD is the most common respiratory disorder among infants born extremely premature. It occurs in ~40% of infants born at less than 28 weeks of gestation and is caused by developmental immaturity which results in inflammation and injury of the lungs accompanied by ineffective repair mechanisms. The fetal lung develops in hypoxic and fluid filled conditions in the uterus, with the low oxygen tension being important for MSCs to retain their normal function during development. Consequently, the hyperoxic treatments used for BPD preterm infants can alter the function of these cells.^74^

The human fetal lung is abundant in LR-MSCs.^75^ LR-MSCs isolated from tracheal aspirates from infants in critical care have been proposed as a biomarker for BPD development.^76^ LR-MSCs were found in 56 out of 84 infants, of which 12 died and 25 developed BPD. Of the remaining 28 infants in which no MSCs were detected, 6 died and 1 developed BPD. Isolation of LR-MSCs from tracheal aspirates was identified as an independent predictor for the development of BPD and may represent a promising biomarker for CLD development.

In rat pups exposed to normoxia or hyperoxia (21% and 95% oxygen, respectively) from birth, isolated LR-MSCs showed divergent differentiation potentials. Normoxic LR-MSCs differentiate along the 3 classical lineages of adipocytes, osteocytes, and chondrocytes. However, hyperoxia-exposed cells produced little to no adipocytes and less osteogenic and chondrogenic matrix.^77^ In addition, hyperoxic LR-MSCs had reduced expression of *fgf-10*, a major determinant in alveologenesis. Similar findings were described in a study using New Zealand white preterm rabbits, whereby short-term hyperoxia (4 h with 50% oxygen) with mechanical ventilation altered the differentiation capacity of LR-MSCs.^78^ After mechanical ventilation, the LR-MSCs exhibited lower adipogenic and osteogenic potentials. In addition, structural analysis using electron microscopy revealed LR-MSCs in the hyperoxia/mechanical ventilation group had evidence of cellular stress in the nucleus, smaller mitochondria, and distended endoplasmic reticula.^78^ More recently, a study of murine LR-MSCs using scRNA-seq revealed that hyperoxia alters their gene signature, with elevated expression in inflammatory, fibrotic, and angiogenic factors.^25^ Interestingly, their analysis revealed that the communication between LR-MSCs driving this gene expression profile in hyperoxia came from immune and endothelial cells.

The in vivo data is consistent with the data from human fetal LR-MSCs and highlights that hyperoxia perturbs the normal function of these cells. In hyperoxic conditions (60% oxygen), LR-MSCs continue to proliferate post-confluence and begin to produce ECM associated with BPD development.^75^ In addition, hyperoxic MSCs secrete minimal amounts of vascular endothelial growth factor (VEGF) compared to those cultured in a lower oxygen environment. Taken together, these findings reveal that hyperoxia and mechanical ventilation, used for the treatment of BPD, alter the function of LR-MSCs via the reduction of their differentiation potential and diminished expression of growth factors involved in homeostasis and repair (Fig. 2D).

### Bronchiolitis Obliterans (BOS)

BOS is characterized by the small airways becoming progressively obstructed leading to persistent airflow limitation. The development of BOS is akin to chronic graft-versus host disease (GVHD), as the immune system attacks the small airways and can occur after lung or hematopoietic stem cell transplantation.

LR-MSCs from patients with BOS are pro-fibrotic and possess markers of myofibroblasts, demonstrating increased α-SMA expression and collagen secretion.^40^ In addition, LR-MSCs in BOS patients are abundant and produce higher levels of endothelin-1, which is known to promote MSC proliferation, migration, and differentiation.^79^ Furthermore, in BOS patients, LR-MSCs secrete less prostaglandin E2 (PGE2) and show resistance to cyclooxygenase-2 (Cox-2) stimulation.^80^ This may be important in the pathogenesis of BOS as PGE2 is anti-inflammatory, and inhibits the proliferation and differentiation of LR-MSCs into myofibroblasts.

More recently, LR-MSCs isolated from the BAL fluid of BOS patients (and compared to stable lung transplant patients) demonstrated deregulated expression of epigenetic enzymes including histone deacetylases (HDAC) -1, -2, -3, and -8, and methyltransferases DNMT1, 3B, and EZH2, as well as several miRNAs.^81^ Both HDACs and methyltransferases have been shown to have a role in fibrotic processes. The pro-fibrotic phenotype of LR-MSCs was confirmed by the upregulation of pro-fibrotic miRNAs (miR-199 family, miR-142-3p) and downregulation of anti-fibrotic miRNAs (miR-145, miR-206, miR-125b, let-7c).^81^ Overall, LR-MSCs appear to have a role in the pathology of BOS via their diminished immunomodulation and promotion of fibrotic processes.

### Asthma

Little is currently known about the role of LR-MSCs in the pathogenesis of asthma. However, one study did identify both higher cell numbers and an increased colony forming unit-fibroblast (CFU-F) capacity in a murine model of allergic airway disease with ovalbumin sensitization and challenge.^82^ Moreover, cells with similar characteristics to LR-MSCs were identified in the BAL fluid from one out of the 3 asthma patients, although further work is required to assess whether there is indeed a pathogenic role for LR-MSCs in asthma.

In summary, the pathomechanisms of LR-MSCs in chronic lung disease appear to be linked to their increased proliferation, altered immunomodulation, impaired regenerative capabilities, and differentiation into myofibroblasts (consequently accounting for the fibrotic aspects).

## Lung Resident MSCs in Pulmonary Repair and Regeneration

It is important to note that during aging the function of MSCs declines.^83^ Over time, ROS and the consequent oxidative stress lead to DNA damage that accumulates, causing cellular aging phenotypes and diminished cellular function.^83^ Although an understanding of the mechanisms of LR-MSC senescence in IPF is lacking, it is known that BM-MSCs from IPF patients are (A) senescent and can induce senescence in neighboring cells via paracrine signaling, (B) have reduced migratory potential, and (C) possess smaller mitochondria than age-matched controls.^84^ Based on this, one could assume that the presence of senescent LR-MSCs in the aged lung would therefore result in a diminished repair capacity and exacerbate chronic lung diseases, such as IPF and COPD. However, a study comparing the phenotypic and functional properties of BM-MSC biobank samples extracted from adult (average age: 38 years) and elderly (average age: 72 years) donors found that in vivo aging had little influence on the cells’ characteristics. Instead, in vitro aging with prolonged culture expansion was shown to impair the regenerative capacities of these cells.^85^ Consequently, the impacts of aging and various disease pathologies on LR-MSCs remain to be determined.

Throughout our lifetime, the lungs are continually exposed to a plethora of damaging stimuli and so require a system that is continually balancing damage with regenerative processes. LR-MSCs are important regulators of repair in pulmonary tissues due to their ability to proliferate and differentiate into fibroblasts to directly participate in the structural repair of a wound.^74^ They also function through direct cell-to-cell and paracrine actions that modify the activation of the surrounding epithelial, endothelial, and immune cells resulting in cytoprotection and repair. Indeed, resident lung stromal cell progenitors have been shown to accumulate in the subepithelial compartment after naphthalene injury, which suggests that they are recruited to participate in the wound healing response.^86^

### Fibroblast Growth Factor 10

FGF-10 can inhibit lung injury and promote lung repair after various stresses including bleomycin, influenza infection, ventilation-induced lung injury, and naphthalene.^87^ In IPF patients, isolated LR-MSCs demonstrated a reduced FGF-10 expression in progressive disease when compared to stable patients.^67^ TGF-β1, a major pro-fibrotic factor relevant to IPF, has been shown to reduce FGF-10 expression in LR-MSCs.^67,88^ HH signaling can also reduce FGF-10 and is involved in epithelial and mesenchymal quiescence to actively maintain postnatal tissue homeostasis, the loss of which leads to aberrant repair mechanisms.^87^ In COPD, like in IPF, LR-MSCs express lower levels of FGF-10.^73^ Loss of this alveolar epithelial mitogen causes a reduced ability to maintain epithelial progenitors and thus repair the damaged epithelium which is a major stimulus for the development of fibrosis.

### Other Paracrine Factors

In a study comparing LR-MSCs from healthy and COPD patients,^73^ LR-MSC-conditioned mediums from both healthy and COPD patients were able to comparably improve wound closure in an epithelial scratch wound assay (using A549 cells), reduce oxidative-stress induced cell damage, and improve the migratory and proliferative responses of A549 cells upon electric field-induced cell death. However, when comparing healthy and COPD LR-MSCs in the alveolosphere organoid model—used for assessment of regenerative capacity, the addition of COPD LR-MSCs to the human lung cell line, NCI-H441, resulted in the formation of larger organoids with lower expression levels of the type II marker, Surfactant protein C (SPC), at earlier stages. Interestingly, organoids derived from unfractionated COPD lung cell suspensions displayed similar abnormalities, forming larger organoids, while organoids generated from EpCAM+ sorted epithelial cells did not display such differences. These findings suggest that differences in organoid formation depend on the dysregulated communication between cells present in unfractionated suspensions (including CD90+ stromal cells). The authors postulated that the lower levels of growth factors released from LR-MSCs, including HGF and FGF-10, may contribute to the impairment of alveolar epithelial regeneration and reduced migration of supportive cells to the damaged site. These findings, using human cells, support previous work using a murine elastase model of emphysema, which revealed that intratracheal administration of LR-MSCs resulted in enhanced HGF expression and the promotion of ATII cell numbers as an MSC-dependent paracrine mechanism for the repair of injured alveoli.^89^ HGF is a mesenchymal-derived paracrine factor that has pleiotropic effects and can promote epithelial proliferation, morphogenesis, migration, and anti-apoptotic responses, thus making it an important mechanism of repair for LR-MSCs.

Other factors which are known to be released by LR-MSCs and have been shown to have functional effects on lung repair include keratinocyte growth factor (KGF) and VEGF. Rat LR-MSCs (isolated from BAL fluid, expanded in vitro, and used as a therapeutic) migrated to inflammatory sites, released KGF, and caused increased SPC expression, indicating stimulation of ATII cell proliferation.^90^ KGF has roles in the differentiation and proliferation of epithelial cells, angiogenesis, and barrier function; therefore, it has been suggested to play a role in pulmonary repair. For repair to occur in the lung, it is important for the tissue to generate a new blood supply via angiogenesis. LR-MSCs express VEGF to promote angiogenesis and facilitate tissue repair; however, under certain pathological settings such as BPD where mechanical ventilation and hyperoxia are used, VEGF release is diminished.^78^

### Repair Through Regulatory T Cells

Another mechanism of repair that LR-MSCs may function through, involves the stimulation of regulatory T (Treg) cells, which secrete factors including KGF and amphiregulin. LR-MSCs have been shown to reduce T-cell proliferation and induce Treg differentiation.^90^ Treg cells promote regeneration in the lungs by secretion of paracrine factors, remodeling of the ECM, and maintaining barrier integrity via coordination with parenchymal cells.^91^

### Mitochondrial Transfer

MSCs have been shown to connect to other cells via tunneling nano-tubules and gap junctions. LR-MSCs can form gap junction communications and transfer cytoplasmic components with alveolar and bronchial epithelia.^92^ Similarly, LR-MSCs can form microtubules and tunneling nanotubes to transfer cytoplasmic components and mitochondria.^57^ As a mitochondrial transfer from BM-MSCs is known to play a role in recovering cellular function and aiding repair, it is hypothesized that LR-MSCs likely function in a comparable manner.

## Concluding Remarks

Lung-resident MSCs are different from MSCs derived from other tissues. They play significant roles in driving the pathogenesis of and promoting regeneration in chronic lung diseases, and so should be considered a target for therapeutic strategies. The behavior of LR-MSCs is altered in multiple lung diseases, often contributing to disease pathogenesis; therefore, these abnormalities should be taken into account when considering autologous LR-MSCs for cell-based therapeutics. Further work on characterizing LR-MSC functionality and means of their communication with other cell types in the lung is required to improve our understanding of the mechanisms of disease and to develop specialized and targeted approaches to treat chronic lung diseases. The use of modern (multi-)omics technologies will greatly facilitate this. Finally, it is conceivable that if considered as a cell therapy for chronic lung disease, LR-MSCs from healthy tissue may provide greater therapeutic efficacy over MSCs derived from other tissue sources, given that they are already trained in the homeostatic mechanisms of the lungs. Finding the MSCs with the most potent effects for specific chronic lung diseases is an exciting area of research with major clinical importance.

## Data Availability

No new data were generated or analyzed in support of this research.
